# Global Health Governance and the WHO Pandemic Agreement: A Scoping Review of Challenges and Analysis of Reforms

**DOI:** 10.3390/ijerph22121807

**Published:** 2025-11-30

**Authors:** Kadria Ali Abdel-Motaal, Wafa Abu El Kheir-Mataria, Sungsoo Chun

**Affiliations:** Institute of Global Health and Human Ecology, American University, New Cairo 11835, Egypt; kkmotaal@aucegypt.edu (K.A.A.-M.); wafamataria@aucegypt.edu (W.A.E.K.-M.)

**Keywords:** pandemic preparedness, global health governance, WHO pandemic agreement, vaccine equity, international health regulations, treaty implementation

## Abstract

Background: The COVID-19 pandemic exposed persistent weaknesses in global health governance, particularly in preparedness, equity, and accountability. The WHO Pandemic Agreement, adopted in May 2025, aims to address these systemic gaps through a binding international framework. Objective: To identify key challenges in global pandemic preparedness and health governance reported in the literature (2019–2024) through a systematic scoping review, and to evaluate how these challenges are addressed in the provisions of the WHO Pandemic Agreement via qualitative document analysis. Methods: Using Joanna Briggs Institute methodology and PRISMA-ScR guidelines, we systematically identified and thematically analyzed 52 peer-reviewed studies published between 2020 and 2024. The thematic results informed a qualitative document analysis of the WHO Pandemic Agreement text to assess the extent to which its provisions address the identified challenges. Results: Persistent gaps in governance (limited enforceability, fragmented coordination), equity (inequitable access to medical countermeasures), capacity (technology transfer and financing), and accountability were identified. Health systems in low- and middle-income countries continue to face critical resource constraints and lack robust mechanisms to ensure accountability and continuous learning. Document analysis showed the WHO Pandemic Agreement addresses coordination and financing but offers limited advances in enforcement, technology transfer, and independent monitoring. Conclusion: The WHO Pandemic Agreement introduces important institutional and financing measures, but persistent gaps remain in enforcement, technology transfer, and inclusive implementation. Strengthening these domains is crucial to achieving equitable and resilient preparedness. By systematically linking evidence from the pandemic preparedness literature to Treaty provisions, this study offers a novel analytical framework for assessing how global health treaties respond to research-identified challenges.

## 1. Introduction

The COVID-19 pandemic exposed critical weaknesses in national health systems and revealed the shortcomings of international preparedness mechanisms, showing that individual countries cannot manage public health emergencies in isolation [1]. The Global Health Security (GHS) Index further confirmed that no country was fully prepared for such a crisis [2]. International governance mechanisms proved ineffective, as many countries adopted nationalist policies that weakened coordinated global responses, underscoring the urgent need for a unified framework to manage future health emergencies [2].

Regional variability in pandemic responses highlights the need for a universal preparedness framework that can be adapted to diverse contexts [3]. While preparedness strategies have historically been reactive, there is now a shift toward proactive approaches emphasizing early intervention to prevent widespread outbreaks. However, conceptual and practical gaps remain in how preparedness is defined and operationalized [4]. The pandemic also spurred numerous initiatives to enhance international collaboration in research, surveillance, and vaccine development, including the Engineering X Pandemic Preparedness Program, the WHO’s Access to COVID-19 Tools (ACT) Accelerator, the Global Research Collaboration for Infectious Disease Preparedness (GloPID-R), and the Coalition for Epidemic Preparedness Innovations (CEPI) [5,6,7]. Despite these efforts, significant shortcomings persisted, including criticisms of the WHO’s delayed response, insufficient coordination among governments, and the inability to ensure equitable access to medical countermeasures [8].

As the leading global health authority, the World Health Organization (WHO) has anchored its pandemic response in the International Health Regulations (IHR), which set out member states’ obligations for managing cross-border health threats [9]. In March 2021, global leaders proposed the establishment of a new international pandemic treaty, initially envisioned as a Framework Convention to enable legally binding commitments on shared principles of preparedness, response, and equity [10]. In May 2025, the WHO Pandemic Agreement was formally adopted as a landmark step to strengthen global cooperation, equity, accountability, and resilience in future health emergencies [11].

This review maps key challenges in global pandemic preparedness and governance and considers the extent to which they are acknowledged in the WHO Pandemic Agreement, with the aim of informing strategies for its effective and equitable implementation.

### 1.1. Review Questions

- What are the key challenges identified in the literature on global pandemic preparedness and global health governance?
- How are these challenges addressed in the WHO Pandemic Agreement (adopted May 2025)?

### 1.2. Objectives

- To identify and synthesize key challenges in global pandemic preparedness and global health governance based on existing literature.
- To examine whether, and to what extent, these challenges are addressed in the WHO Pandemic Agreement, noting strengths, omissions, and areas of limited clarity.
- To propose evidence-informed recommendations to support the effective and equitable implementation of the WHO Pandemic Agreement.

## 2. Materials and Methods

### 2.1. Rationale and Study Design

This study used a mixed qualitative design combining a scoping review and a document analysis. The aim was to identify key challenges in global pandemic preparedness and global health governance, and to examine how these challenges are addressed in the WHO Pandemic Agreement adopted on 20 May 2025. The two components are complementary: the scoping review mapped the breadth of peer-reviewed and gray literature (2019–2024) to synthesize evidence on governance challenges, while the document analysis examined how the treaty text reflects or responds to these identified issues. This dual approach follows established guidance from the Joanna Briggs Institute (JBI) and the PRISMA-ScR framework for evidence mapping, combined with accepted qualitative techniques for policy and treaty content analysis. Together, they enable triangulation between evidence-based challenges and the formal provisions of a newly adopted global legal instrument.

### 2.2. Scoping Review

Given the breadth and conceptual diversity of literature on pandemic preparedness and global health governance, the study adopted a scoping review design. Unlike systematic or narrative reviews that aim to evaluate study quality or test specific hypotheses, scoping reviews are intended to map the range and characteristics of available evidence, particularly across complex and interdisciplinary policy domains. This approach enabled comprehensive synthesis of evidence spanning legal, policy, and health systems perspectives, without restricting inclusion by study design or outcomes. Following the Joanna Briggs Institute (JBI) methodology and the PRISMA-ScR (Preferred Reporting Items for Systematic Reviews and Meta-Analyses extension for Scoping Reviews) guidelines, the review systematically characterized the nature and extent of available evidence to inform the subsequent document analysis of the WHO Pandemic Agreement. Consistent with PRISMA-ScR recommendations, critical appraisal and data-code availability were not undertaken, as these are optional components for scoping reviews, focused on evidence mapping rather than quality assessment (Appendix A).

A broad and systematic literature search was conducted across major electronic databases: PubMed, Scopus, Web of Science, and in the academic search engine, Google Scholar. In addition, targeted searches of gray literature were performed on websites of key international organizations, including the World Health Organization (WHO), Centers for Disease Control and Prevention (CDC), and United Nations (UN), to capture relevant policy documents, reports, and briefs. The search incorporated both academic and practitioner perspectives to encompass the political and governance dimensions of the topic.

#### 2.2.1. Search Strategy and Screening

The search strategy was developed in accordance with JBI recommendations, combining Boolean operators, free-text keywords, and controlled vocabulary to maximize sensitivity. For example, in PubMed, Medical Subject Headings (MeSH) such as “Pandemics” [MeSH] were paired with free-text terms like “pandemic preparedness” [Title/Abstract] to capture both indexed and non-indexed records. A systematic search was then conducted across selected databases, with search strings tailored to the syntax of each. The full strategies for all databases are provided in Appendix B. For illustration, the PubMed search string was:

(“pandemic preparedness” [Title/Abstract] OR “global health governance” [Title/Abstract] OR “international health regulations” [Title/Abstract] OR “pandemic treaty” [Title/Abstract] OR “pandemic agreement” [Title/Abstract]) AND (“2019/01/01” [Date—Publication]: “2024/05/01” [Date—Publication])”

#### 2.2.2. Inclusion and Exclusion Criteria

This targeted approach applied inclusion and exclusion filters defined hereinafter.

##### Inclusion Criteria

Publications focusing on legal, institutional, or governance challenges related to pandemic preparedness treaties or frameworks; Analyses of global health governance mechanisms published post-2019; Peer-reviewed publications and gray literature in English, including articles, reviews, reports, policy briefs, commentaries, editorials, and other relevant formats that analyze legal, institutional, or governance aspects of pandemic preparedness.

##### Exclusion Criteria

Studies not directly addressing aspects of pandemic preparedness or international health legal instruments; Publications dated prior to 2019 (with justified exceptions); non-English language sources (due to resource constraints).

#### 2.2.3. Study Selection

Three independent reviewers conducted a two-stage screening process involving title and abstract review, followed by full-text assessment of potentially eligible articles. Any disagreements were discussed among the three reviewers until a consensus was reached. The reviewers were selected for their expertise in global health governance and public health systems, as well as prior experience with review methodology, which ensured their ability to make informed and reliable decisions about article eligibility.

#### 2.2.4. Timeline

The scoping review was undertaken over five months, from 1 May 2024 to 31 October 2024 and is contextualized against the subsequent adoption of the WHO Pandemic Agreement in May 2025. It is structured as follows:

- Planning and protocol development: 1 month
- Literature search and screening: 1.5 months
- Data extraction and synthesis: 1.5 months
- Reporting and manuscript preparation: 1 month

#### 2.2.5. Data Extraction and Charting

Data extraction was performed using a standardized form in the Covidence platform (Covidence is a systematic review management tool designed to streamline citation screening, full-text review, data extraction, and quality assessment). Three reviewers independently screened titles and abstracts for relevance, followed by full-text reviews. Discrepancies were resolved by consensus. Two studies published prior to 2019 were retained due to their conceptual importance [12,13]. The first one was among the earliest studies to define and establish global health diplomacy as a critical, growing field linking health with foreign policy, security, and international cooperation, a key context for understanding pandemic treaty governance. The second one is a thorough analysis of the International Health Regulations (IHR), the central legal framework for global health security, essential for assessing governance and reform needs before COVID-19. The key information extracted from each publication included publication type, country, and date, thematic focus (e.g., governance, equity), and main findings related to pandemic preparedness and response. Data were organized into a standardized Excel matrix to facilitate thematic analysis and mapping of evidence.

### 2.3. Document Analysis of the WHO Pandemic Agreement

In addition to the scoping review, a focused document analysis of the WHO Pandemic Agreement (WHA78.1), adopted on 20 May 2025, was conducted. The official text, including available annexes such as the draft Pathogen Access and Benefit-Sharing (PABS) Annex, was downloaded from the WHO website on 10 June 2025 and treated as a primary policy document for qualitative content analysis.

Themes generated by the scoping review served as an a priori coding framework. Two independent reviewers identified and coded treaty provisions that (i) directly addressed, (ii) partially addressed, or (iii) omitted each challenge area. Draft annexes still under negotiation at the time of analysis were coded as Pending. Consistent with established qualitative policy research standards, two independent reviewers coded treaty provisions to improve reliability and resolve subjective coding judgments. Discrepancies were discussed until consensus was reached, ensuring transparency and rigor in the mapping process. Data were summarized in a matrix mapping each literature-identified challenge to the corresponding provisions of the WHO Pandemic Agreement (WHA78.1), adopted on 20 May 2025 (Appendix C). This annex presents the results of the document analysis; cross-referencing governance challenges identified in the scoping review with specific treaty articles. Each entry indicates whether the Agreement fully addresses, partially addresses, omits, or defers (Pending) each challenge area. This mapping constitutes an integrated evidence-to-policy assessment framework, enabling systematic evaluation of how the WHO Pandemic Agreement operationalizes key governance challenges and supporting future monitoring of its implementation and accountability.

This approach follows established guidance for policy and treaty content analysis ensuring transparency and methodological rigor. By aligning literature-based evidence with treaty provisions, this method enables an interpretive understanding of how the Pandemic Agreement acknowledges or responds to key governance challenges identified in the recent global health discourse.

## 3. Results

The results are organized around the four thematic domains identified through the scoping review: (1) global health governance and the WHO Pandemic Agreement, (2) vaccine equity and intellectual property barriers, (3) preparedness infrastructure and monitoring mechanisms, and (4) lessons learned from the COVID-19 pandemic. For each theme, findings from the document analysis of the WHO Pandemic Agreement are presented alongside the corresponding literature-identified challenges, illustrating how specific treaty provisions address, or fail to address, these issues. This integrated presentation demonstrates the complementarity of the two analytical phases and provides a coherent framework for evaluating the Agreement’s capacity to operationalize global health governance.

### 3.1. Results of the Scoping Review

**Figure 1:**
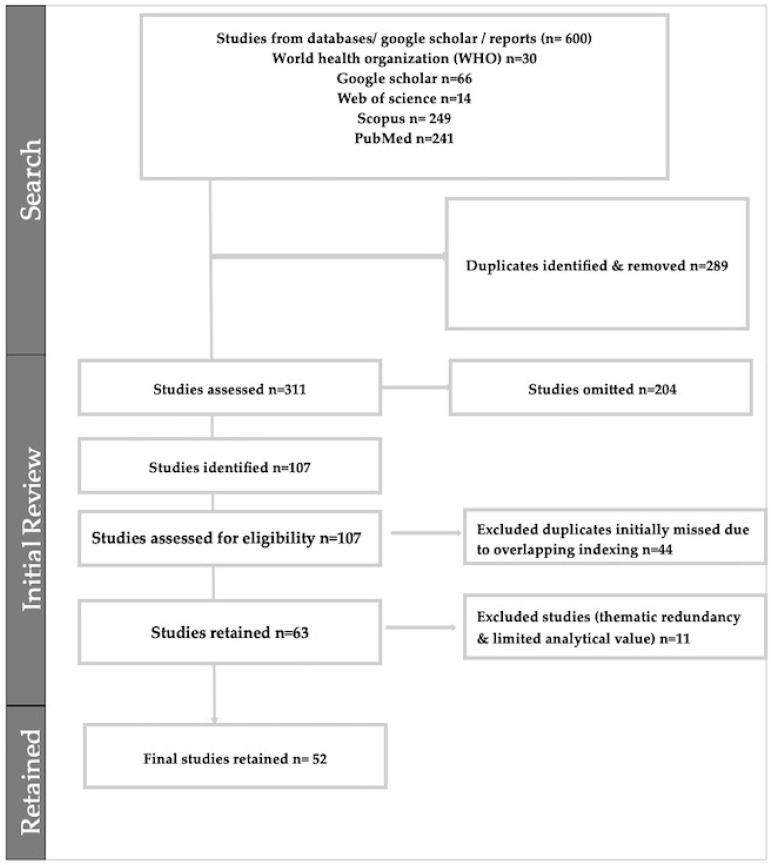
Prisma Flow Diagram.

The initial search across the four selected electronic databases and gray literature sources yielded 600 records. After the first round of duplicate removal, 311 unique records remained for title and abstract screening. During this stage, 204 records were excluded for not meeting the inclusion criteria. A further 44 duplicates, initially missed due to overlapping indexing across databases, were also identified and removed at this point. This left 63 full-text articles for eligibility assessment. Eleven studies were excluded during the data extraction phase due to thematic redundancy and limited analytical value. Finally, 52 studies met the inclusion criteria and were included in the final synthesis. The full study selection process is presented in the PRISMA-ScR flow diagram (Figure 1).

### 3.2. Classification of the Studies

The included studies were classified according to type, country focus, and year of publication to provide a contextual overview of the evidence base.

The study types, including empirical research, policy analyses, conceptual papers, and reviews, are displayed in Table 1. The studies categorized by their geographical settings are displayed in Table 2. While Table 3 summarizes the temporal distribution of the studies included in this review, showing publication patterns across the years 2020–2024.

### 3.3. Thematic Analysis

An inductive thematic analysis was conducted to systematically organize and interpret the extracted data. Initially, open coding was applied to generate preliminary concepts, which informed the development of higher-order analytical categories. This was followed by focused coding to identify patterns, relationships, and recurring challenges across the literature. The analysis yielded four overarching thematic domains:

1. Global health governance and the proposed Pandemic Treaty
2. Vaccine equity and intellectual property barriers
3. Preparedness infrastructure and monitoring mechanisms
4. Lessons learned from the COVID-19 pandemic

These themes provide a structured lens to examine key challenges and opportunities in advancing effective and equitable pandemic preparedness and response.

#### 3.3.1. Theme One: Global Health Governance and the WHO Pandemic Treaty

This theme synthesizes findings from twenty-six studies focusing on nine interconnected subthemes that collectively address the multifaceted challenges, priorities, and reform opportunities within global health governance and how they are addressed in the new pandemic treaty (Table 4).

##### Global Health Security and Universal Health Coverage

The literature highlights a persistent structural tension between the Global Health Security (GHS) paradigm, which prioritizes the rapid containment of cross-border health threats, and the Universal Health Coverage (UHC) approach, which emphasizes sustained investment in equitable, people-centered health systems [14,15,16]. In practice, GHS often privileges surveillance, early detection, and emergency response, typically coordinated by centralized authorities. Critics argue that this security-oriented focus sidelines social determinants of health and diverts resources away from primary health care, especially in low-resource settings [15]. By contrast, UHC is anchored in principles of equity and access through health system strengthening, yet it has been critiqued as insufficiently agile in responding to acute public health emergencies when pursued in isolation from GHS [16]. The COVID-19 pandemic underscored the limitations of relying exclusively on either paradigm: countries with strong emergency response capacities but underdeveloped primary care, as well as those with robust primary care but limited preparedness systems, both struggled to maintain effective pandemic control. Scholars therefore emphasize that integrating GHS and UHC is not simply complementary but essential for developing resilient, equitable, and sustainable preparedness architectures [17].

##### Equitable Access to Medical Countermeasures

The COVID-19 pandemic underscored persistent inequities in access to medical countermeasures, particularly vaccines and antivirals, with LMICs often receiving them months after high-income countries (HICs), if at all. The COVAX initiative (COVAX is the vaccines pillar of the Access to COVID-19 Tools (ACT) Accelerator, co-led by Gavi, the Vaccine Alliance, the World Health Organization (WHO), and the Coalition for Epidemic Preparedness Innovations (CEPI)), though intended to ensure global equity, delivered only 16% of the doses originally planned for LMICs by mid-2021, revealing deep structural limitations in voluntary international cooperation mechanisms [18]. Several studies attribute these failures to intellectual property barriers, limited local manufacturing capacity in the Global South, and the prioritization of bilateral deals by HICs that bypassed multilateral distribution platforms [18]. Without enforceable global commitments or mechanisms for redistribution, structural inequalities in pandemic response remain entrenched.

##### Subsidiarity, Solidarity, and Decolonization in Health Governance

The reviewed studies emphasize that effective pandemic governance should be grounded in the principle of subsidiarity, whereby decisions are made at the lowest level capable of addressing a given issue [19,20]. Yet, the WHO Pandemic Agreement largely reflects a top-down, state-centric orientation, with limited formal recognition of the roles of regional institutions and municipal health authorities. For example, although the Africa Center for Disease Control and Prevention (Africa CDC) demonstrated substantial capacity in coordinating cross-border responses during COVID-19, its integration into global treaty structures remains marginal. Scholars further caution that the treaty’s current framing risks reproducing unequal power relations by positioning low- and middle-income countries (LMICs) primarily as beneficiaries of aid rather than as active co-creators of governance solutions [19,20]. This dynamic is particularly salient in negotiations over the Pathogen Access and Benefit-Sharing (PABS) mechanism, where LMICs have historically contributed biological samples but received limited reciprocal benefits in the form of vaccines, diagnostics, or research outputs. Without explicitly incorporating decolonial perspectives and equity safeguards, the treaty risks undermining both legitimacy and trust, especially in regions with long-standing concerns about extractive global health practices [19].

##### Global Health Diplomacy and Treaty Negotiations

The literature underscores global health diplomacy as a vital tool for navigating competing national interests in multilateral treaty-making processes, particularly under conditions of geopolitical fragmentation [12,21,22,23]. During the COVID-19 pandemic, diplomatic mechanisms such as COVAX aimed to promote equitable vaccine distribution, yet ultimately exposed the limits of voluntary cooperation, as wealthier nations prioritized bilateral deals and vaccine stockpiling, thereby undermining the collective goals of equitable access and global solidarity [24]. Parallel analyses show that broader treaty negotiations remain constrained by sovereignty concerns, particularly among high-income countries wary of ceding decision-making authority to international bodies [25]. This tension has led to diluted language around compliance mechanisms and legal accountability, weakening the treaty’s enforceability [26]. Moreover, the persistence of “vaccine nationalism” in treaty deliberations has raised concerns about the feasibility of securing binding commitments to resource sharing during future crises. Scholars highlight the need for formalized dispute resolution mechanisms and equitable representation of LMICs in global health decision-making forums to overcome existing power asymmetries and foster durable cooperation [26].

##### The Role and Limitations of the International Health Regulations (IHR)

The IHR, developed in 2005, serves as the primary legal instrument for global health emergency coordination. However, their reliance on non-binding recommendations, lack of enforcement mechanisms, and underfunded implementation capacities have limited their effectiveness during the COVID-19 pandemic [13,27,28]. Several studies point out that during the early phases of the outbreak, delays in state reporting, the absence of standardized risk communication, and inconsistent application of travel restrictions demonstrated the IHR’s operational limitations [27]. Moreover, the WHO’s authority to verify information and issue alerts was constrained by state sovereignty and political sensitivities, which undermined the IHR’s intent to provide transparency and early detection [28]. As a result, scholars and policy analysts advocate for a new legally binding pandemic treaty that strengthens reporting obligations, includes independent verification protocols, and introduces enforcement mechanisms such as compliance monitoring or sanctions [29]. A key recommendation is the establishment of a Conference of the Parties (COP) to oversee implementation, ensure periodic review of commitments, and provide a formal structure for accountability, functions that are not currently embedded within the IHR framework [29].

##### Deep Prevention and Multisectoral Risk Reduction

The concept of deep prevention emphasizes proactive measures aimed at mitigating the root causes of pandemics, including deforestation, wildlife trade, antimicrobial resistance, and underinvestment in primary health systems [30]. Rather than focusing solely on emergency outbreak responses, this approach promotes long-term structural interventions to reduce zoonotic spillovers and environmental triggers. Reviewed studies strongly advocate for integrated, multisectoral strategies aligned with the *One Health* framework, which links human, animal, and environmental health systems. However, implementation remains limited due to fragmented institutional mandates, inadequate cross-sectoral coordination, and insufficient political will, particularly in low-resource settings [30].

##### Treaty Design and Multi-Level Governance

The literature underscores the importance of aligning treaty design with the realities of multilevel governance in global health, where responsibilities are distributed across local, national, and global levels. At the local level, this involves frontline service delivery and community engagement; at the national level, policymaking, resource allocation, and health system leadership; and at the global level, standard-setting, coordination, and oversight. Scholars emphasize that effective pandemic governance requires clear delineation of these roles to ensure coherence, accountability, and timely response [31].

Studies highlight the risks of fragmented governance in the absence of coordination mechanisms between global, national, and sub-national authorities, especially in decentralized health systems where alignment across levels is essential [32]. In response, experts in global health law call for governance frameworks that clearly assign responsibilities across global, national, and regional levels, establish independent mechanisms to monitor compliance, and allow regional adaptation of measures while ensuring alignment with overarching global standards [31,32].

##### Public Health Priorities for Pandemic Resilience

The literature consistently highlights several core priorities for strengthening pandemic resilience, including: (i) reinforcing primary health care systems, (ii) ensuring resilient and diversified supply chains, and (iii) expanding equitable access to vaccines, diagnostics, and therapeutics [33,34,35,36]. Multisectoral coordination is emphasized as essential for aligning efforts across health, environment, and economic sectors, particularly in implementing the One Health approach [33,34]. Scholars also emphasize the need for stronger accountability mechanisms and clearly defined compliance benchmarks to assess state performance, as persistent ambiguity in reporting standards undermines effective international monitoring and enforcement [27,35,36].

##### Future Pathways and Structural Reforms

The literature proposes several structural reforms aimed at enhancing long-term pandemic preparedness and equitable access to medical countermeasures. These include establishing regional research and development hubs to diversify knowledge production, particularly in LMICs, and investing in decentralized manufacturing infrastructure to reduce dependence on high-income countries during health emergencies [37]. Additionally, researchers call for the reform of intellectual property (IP) regimes, specifically advocating for mechanisms that facilitate compulsory licensing, knowledge sharing, and technology transfer agreements to ensure broader access to life-saving tools during pandemics [37]. The failure to implement such reforms, authors warn, may perpetuate current inequalities and significantly limit the effectiveness of any global pandemic governance framework.

In summary, this theme synthesizes evidence from 26 studies showing that global health governance remains characterized by structural imbalances and unresolved tensions between security-driven and equity-driven approaches. Voluntary cooperation mechanisms have proven insufficient, and gaps in intellectual property governance, benefit-sharing, and regional integration persist. Collectively, these findings point to the need for a binding, multi-layered governance instrument that clarifies responsibilities across governance levels, ensures meaningful participation of LMICs, and embeds principles such as One Health into enforceable frameworks.

##### How Are These Challenges Addressed in the WHO Pandemic Agreement [37,38]

The WHO Pandemic Agreement addresses several of the governance challenges identified in theme one, though with mixed effectiveness. It reinforces transparency and timely information-sharing by establishing binding obligations for reporting on outbreaks, public health capacities, and compliance with core requirements (Articles 3, 4, and 11). The Agreement establishes obligations for sustainable financing, including the creation of a Coordinating Financial Mechanism and the promotion of innovative financing measures through bilateral, regional, and multilateral channels, with the aim of supporting equitable access for low- and middle-income countries (Articles 13 and 18). Legal accountability is reinforced through the establishment of governance structures such as the Conference of the Parties, mandated to oversee implementation and compliance (Articles 17 and 31). In parallel, the Agreement advances multisectoral prevention by embedding the One Health approach, requiring integration across human, animal, and environmental health systems (Articles 5 and 12). However, the agreement does not explicitly empower local or regional authorities to make context-specific decisions, limiting the principle of subsidiarity and potentially reducing responsiveness at subnational levels. Furthermore, commitments to guaranteed funding remain vague, enforcement mechanisms are weak, and provisions related to intellectual property rights are underdeveloped, posing significant challenges to ensuring equitable access and effective implementation. Mechanisms for data governance and operational equity targets also lack clarity. The key components of the Pathogen Access and Benefit Sharing (PABS) Annex are still under negotiation, with finalization expected by the 2026 World Health Assembly [37,38].

To summarize, the WHO Pandemic Agreement introduces significant governance innovations; however, it leaves critical gaps unaddressed. These omissions risk perpetuating inequities and governance limitations identified in the literature, suggesting that while the treaty introduces ambitious provisions, it does not fully resolve the structural and systemic challenges of global health governance.

#### 3.3.2. Theme Two: Vaccine Equity and Intellectual Property Barriers

This theme synthesizes findings from six studies focusing on three interconnected subthemes: equitable vaccine distribution challenges, intellectual property barriers to vaccine technology access, and mechanisms for operationalizing equity within the WHO Pandemic Accord (Table 5).

##### Challenges in Vaccine Distribution and Equity

The COVID-19 pandemic exposed profound inequities in global vaccine allocation, as low- and middle-income countries (LMICs) experienced prolonged delays in access and substantially lower coverage compared to high-income countries. These disparities stemmed from the concentration of vaccine production capacity in high-income countries, insufficient technology transfer to LMICs, and the dominance of bilateral purchasing agreements that undermined multilateral distribution initiatives such as COVAX [39]. Although the WHO’s Immunization Agenda 2030 advocates for enhancing vaccine research, development, and production capacities in LMICs, progress remains slow due to resistance from wealthier nations over issues such as intellectual property waivers and profit-sharing [39]. Despite the efforts towards the universal acknowledgment of the moral and public health imperative for equitable access, the literature shows no consensus on the governance model necessary to implement such equity in future pandemics [39].

##### Intellectual Property Barriers to Vaccine Technology Access

Restrictive intellectual property (IP) frameworks have been identified as a central barrier to timely and equitable access to vaccine technologies in LMICs [40]. Specific obstacles included patent protections on key vaccine components, proprietary control over production methods, and the non-disclosure of manufacturing know-how, such as trade secrets, all of which constrained LMIC producers from scaling up local manufacturing during COVID-19 [40]. Although the TRIPS Agreement permits compulsory licensing as a legal flexibility, its application has been limited in practice due to procedural complexity, diplomatic opposition from high-income countries, and the risk of trade retaliation [41]. In response, some scholars propose hybrid models that combine patent protection with compulsory licensing of undisclosed technologies, seeking to balance innovation incentives with the need for rapid technology transfer and equitable access during global health emergencies [41]. Yet, these approaches have gained little traction amid geopolitical resistance and industry concerns about undermining intellectual property rights. The limited effectiveness of mechanisms such as COVAX further underscores how deficiencies in global IP governance constrained equitable vaccine distribution, pointing to the need for enforceable multilateral provisions that institutionalize technology transfer and strengthen regional manufacturing capacity [42].

##### Operationalizing Equity in the Pandemic Accord

The WHO Pandemic Agreement introduces Access and Benefit-Sharing (ABS) mechanisms aimed at linking the timely sharing of pathogens with the equitable distribution of resulting vaccines and treatments. However, multiple analyses have shown that past ABS frameworks, such as the Nagoya Protocol and the Pandemic Influenza Preparedness (PIP) Framework, have struggled to achieve meaningful equity in practice, due to vague obligations, lack of enforcement, and minimal financial returns for contributing countries [43,44]. Scholars argue that the effectiveness of the new ABS system depends on embedding enforceable mechanisms, such as automatic IP waivers for pandemic-related technologies, transparent data sharing obligations, and pre-negotiated financing for global distribution [44]. In this context, initiatives like European Union vaccine diplomacy are cited as illustrative cases that combine treaty-based principles with practical tools such as voluntary licensing, local manufacturing partnerships, and sustained capacity building support in LMICs [21].

In summary, the literature converges on three persistent obstacles to vaccine equity: structural imbalances in global production, intellectual property restrictions limiting technology transfer, and the weakness of multilateral governance mechanisms. Attempts to remedy these through Access and Benefit-Sharing (ABS), technology transfer, and funding schemes remain largely aspirational. Taken together, these findings point to the urgent need for enforceable and adequately resourced global frameworks capable of addressing systemic inequities in access to medical countermeasures.

##### How Are These Challenges Addressed in the WHO Pandemic Agreement

The WHO Pandemic Agreement seeks to address critical governance challenges in vaccine equity and intellectual property, though with mixed effectiveness. It establishes a Pathogen Access and Benefit-Sharing (PABS) system to link the rapid sharing of pathogen samples and genetic sequence data with equitable access to vaccines, diagnostics, and therapeutics (Article 10, Article 18). The treaty further underscores the principle of equitable and timely access by creating a dedicated global supply system and allocation mechanism (Article 12), alongside provisions for sustainable financing to support low- and middle-income countries in scaling manufacturing and distribution capacities (Article 13) [37,38].

To summarize, important constraints persist. Intellectual-property provisions rely on existing TRIPS flexibilities and voluntary licensing, with no compulsory licensing triggers or enforceable transfer of manufacturing know-how. PABS modalities (benefit types, allocation rules, trigger conditions) are deferred to future COP decisions, leaving implementation parameters unspecified. Financing obligations are not assessed or binding, and the Coordinating Financial Mechanism’s resourcing and governance are undefined. Capacity-building clauses are framed in promotional terms without quantified benchmarks or timelines, and compliance is routed through COP oversight without sanctions. Absent binding obligations on technology transfer, predictable financing, and measurable allocation targets, these provisions may be insufficient to prevent the distributional shortfalls observed during COVID-19.

#### 3.3.3. Theme Three: Preparedness Infrastructure and Monitoring Mechanisms

This theme synthesizes evidence from thirteen studies addressing seven sub-themes essential to strengthening global preparedness capacity and institutional response (Table 6).

##### Independent Monitoring and Compliance

Scholars emphasized the critical role of independent monitoring in ensuring state compliance with the WHO Pandemic Treaty. They propose the establishment of an autonomous oversight body, equipped with the authority to conduct periodic assessments of national preparedness and treaty adherence. Such a mechanism is viewed as essential for transitioning the treaty from a politically negotiated text into an operational legal framework with enforceable obligations [45].

##### Impact on Least Developed Nations (LDNs)

Structural vulnerabilities, such as chronic underfunding of health systems, reliance on external supply chains, and limited negotiating capacity, continue to place LDNs at a systemic disadvantage within global health governance systems. Addressing these inequities requires treaty provisions that move beyond aspirational language, which currently relies on non-binding commitments, toward enforceable obligations. Such provisions should guarantee equitable access to medical countermeasures, safeguard national sovereignty by ensuring that LDNs retain agency in defining their health priorities and institutionalize mechanisms for sustained participation, such as guaranteed representation in COP deliberations or structured regional consultation platforms [46].

##### Health Systems Resilience and Preparedness

Studies widely recognize the importance of standardized performance indicators for assessing and strengthening health system resilience [47]. Proposed measures include: (i) the development of universal metrics, such as indicators of access, equity, and health outcomes; (ii) the integration of population health management approaches that align services with community needs through preventive care, risk stratification, and coordinated delivery; and (iii) sustained investment in health infrastructure to address inequities across income settings [33]

##### Intellectual Property Rights and Access to Resources

The rigidity of intellectual property regimes during public health emergencies is widely recognized as a major obstacle to equitable access to essential technologies [48]. Scholars advocate for legal reforms that elevate public health priorities over commercial interests, particularly in the context of global crises.

##### Civil Society Engagement

Civil society organizations (CSOs) are recognized as critical actors in translating global health policies into locally effective interventions [49]. Their participation enhances the legitimacy, cultural relevance, and community acceptance of preparedness and response strategies. Scholars emphasize that the exclusion of CSOs from decision-making processes undermines the responsiveness and equity of health systems, particularly in marginalized or high-risk communities [49].

##### Simulation and Preparedness Exercises

The operational value of simulation and preparedness exercises in strengthening pandemic readiness and system-wide coordination was emphasized [50]. These simulations serve as structured opportunities to evaluate response protocols, decision-making chains, and intersectoral communication under real-world conditions. For example, post-COVID analyses noted that countries with institutionalized simulation programs, such as Singapore and South Korea, were able to activate emergency operations faster and with fewer coordination failures [50]. Moreover, cross-border simulations have revealed recurring weaknesses in regional data-sharing and resource mobilization, underscoring the need for standardized scenario planning across jurisdictions. Scholars argue that embedding such exercises into national preparedness strategies can transform static plans into dynamic, actionable frameworks for crisis response [50].

##### Equity and Governance in the Pandemic Treaty

The literature highlights the need for a robust global governance system to ensure equitable access to vaccines, therapeutics, and diagnostics, particularly for low- and middle-income countries (LMICs) [39]. A thematic analysis of 43-member state submissions during treaty negotiations identified equity and justice as widely recognized foundational principles. However, support for adaptive governance mechanisms, such as iterative implementation, localized decision-making, and flexible oversight, was more limited, reflecting ongoing geopolitical and institutional divergence on treaty operationalization [51]. Several scholars argue that strengthening the International Health Regulations (IHR) may offer a more politically viable and expedited pathway than negotiating a new treaty, given the complexity and length of treaty processes [52,53]. This concern is reinforced by evidence that protracted negotiations risk diverting attention from urgent preparedness needs, underscoring the importance of complementary short-term and flexible governance measures [54]. At the same time, critics emphasize that treaty drafts often rely on aspirational language rather than embedding ethical principles into binding commitments with clear enforcement mechanisms [55]. The proposed Access and Benefit-Sharing (ABS) system illustrates this challenge: while conceptually promising, legal experts caution that its overlap with existing international agreements, such as the Convention on Biological Diversity and the IHR, risks creating legal ambiguities and coordination difficulties [56].

In summary, the reviewed literature points to persistent structural deficiencies in global pandemic preparedness that extend beyond treaty design. Priority recommendations include creating independent compliance mechanisms to strengthen accountability, adopting standardized metrics to evaluate health system resilience, and ensuring the meaningful participation of least developed nations (LDNs) in decision-making processes. Scholars also highlight the critical role of civil society in facilitating culturally responsive, community-trusted interventions, while emphasizing the value of institutionalized simulation exercises to test preparedness strategies and decision-making under realistic conditions.

##### How Are These Challenges Addressed in the WHO Pandemic Agreement

The WHO Pandemic Agreement addresses several governance challenges through specific treaty provisions. Articles 19–22 establish a compliance mechanism under the authority of the Conference of the Parties (COP), designed to oversee implementation and promote accountability. This mechanism permits the use of state self-reporting, peer and expert assessments, and external inputs, although its operational independence and enforcement powers remain limited.

Support for Least Developed Nations (LDNs) is explicitly referenced in Articles 8 and 15, which provide for targeted financial and technical assistance to strengthen health systems, expand equitable access to countermeasures, and reduce structural vulnerabilities. Article 6 further obligates states to develop and maintain core public health capacities, including essential health services, preparedness planning, and surveillance, monitored through Joint External Evaluations (JEEs) and National Action Plans for Health Security, with support from designated financial instruments.

Intellectual property challenges are addressed indirectly through the ongoing negotiation of the Pathogen Access and Benefit-Sharing (PABS) system (Article 10 and draft Annex). While this system seeks to link pathogen data sharing with equitable access to vaccines, diagnostics, and therapeutics, critical issues remain unresolved, including licensing triggers, benefit-allocation rules, and enforceable technology-transfer mechanisms.

Operational provisions (Articles 12, 13, and 18) include commitments to conduct simulation exercises, facilitate civil society participation, and strengthen regional manufacturing capacity. However, these clauses often lack binding obligations, concrete benchmarks, or dedicated financing guarantees, leaving implementation dependent on political will and voluntary compliance.

To summarize, the Agreement makes meaningful progress by embedding compliance mechanisms, strengthening LDN participation, and mandating the development of core preparedness capacities. Yet, unresolved enforcement provisions, incomplete intellectual property reforms, and the absence of guaranteed financing weaken its capacity to deliver equity in practice. The PABS Annex remains under negotiation, creating uncertainty around benefit-sharing and access commitments. Similarly, provisions on simulation exercises and civil society engagement remain broadly aspirational, without detailed procedures or sanction-backed accountability. Unless these gaps are addressed, particularly in politically contested domains such as governance design and IP, the Agreement risks replicating the same structural inequities and coordination failures that hampered the COVID-19 response.

#### 3.3.4. Theme Four: Lessons from the COVID-19 Pandemic

Seven studies explored four interconnected subthemes: Critical Gaps in Pandemic Response, missed opportunities, impacts on low- and middle-income countries (LMICs), and the role of regional collaboration. (see Table 7).

##### Critical Gaps in Pandemic Response

The literature identifies several critical deficiencies in global pandemic response systems. Key issues include delayed outbreak reporting, insufficient containment protocols, and inadequate international support for low- and middle-income countries (LMICs), particularly in terms of equitable access to diagnostics, therapeutics, and vaccines [57]. These weaknesses were further exacerbated by widespread misinformation, inconsistent enforcement of public health measures, and marked disparities in health outcomes across countries. Studies emphasize the need for resilient health systems with integrated vaccination infrastructure, strong surveillance mechanisms, and universal health care access. A recurring recommendation is the adoption of a One Health framework, which integrates human, animal, and environmental health domains to strengthen detection and response capacities [58]. Complementary lessons highlight the importance of proactive investments in public health infrastructure, strategic stockpiling of medical countermeasures, research and development, and transparent communication strategies. Together, these measures are recognized as essential components of a globally coordinated response that fosters trust, ensures timely information sharing, and enhances accountability.

##### Missed Opportunities in Workforce and Education

The review underscores a significant gap in medical and public health education, revealing that most medical curricula remain heavily centered on clinical care at the expense of training in essential public health domains, including epidemiology, health systems management, preventive medicine, and population health. Several studies argue that this narrow focus left health professionals underprepared for the system-level challenges posed by COVID-19 [59]. Key areas identified for curricular reform include the integration of disease modeling, epidemiological surveillance, triage protocols during surge capacity (i.e., guidelines for prioritizing care when resources are overwhelmed), digital health tools, and predictive analytics. These competencies are considered essential not only for pandemic preparedness but also for strengthening real-time decision-making and intersectoral coordination in future health emergencies [60].

##### Impacts on LMICs

Low and middle-income countries (LMICs) experienced a dual reality during the COVID-19 pandemic, exacerbated vulnerabilities due to limited infrastructure and funding, alongside emerging opportunities to advocate for a more equitable global health order. The literature critiques governance models that have historically treated LMICs as passive recipients of aid. Instead, scholars emphasize the need for a restructured global health architecture grounded in principles of justice, mutual trust, and shared decision-making authority. Additionally, to recognize LMICs as active agents in pandemic preparedness, to support their regional leadership, and to strengthen their capacity for research and production [61].

##### Regional Coordination

Inadequate regional coordination emerged as a major weakness in pandemic responses. In the Middle East and North Africa (MENA) region, the absence of robust collaborative mechanisms hindered both health and security outcomes [62]. In Southeast Asia, the “Association of Southeast Asian Nations, ASEAN” facilitated limited information exchange but failed to implement a unified regional response strategy. Structural barriers, including socioeconomic disparities and chronic underfunding of health systems, further undermined regional effectiveness. These gaps highlight the need for stronger multi-sectoral coordination, standardized communication protocols, and structural reforms to enhance regional preparedness and response capacities [63].

In summary, the reviewed studies highlight persistent weaknesses in global pandemic response systems, including delays in outbreak detection, inconsistent implementation of public health measures, and inequitable access to medical countermeasures for LMICs. Limited integration across health, environmental, and animal sectors underscored the need for a One Health approach, while gaps in education and workforce training, particularly in epidemiological modeling and digital health, further constrained preparedness. LMICs were disproportionately affected but also emerged as advocates for equity and leadership in global governance. Finally, weak regional coordination, especially in MENA and Southeast Asia, revealed systemic barriers to collaboration, underscoring the need for stronger multisectoral and intergovernmental mechanisms.

##### How Are These Challenges Addressed in the WHO Pandemic Agreement

The WHO Pandemic Agreement incorporates lessons from COVID-19 through provisions that strengthen global and regional coordination under a multisectoral One Health framework, integrating human, animal, and environmental health surveillance and response (Articles 5, 12) [38]. It mandates timely outbreak notification and transparent data sharing to address prior delays and misinformation (Article 10) [38] and prioritizes equitable access for LMICs through financial and technical assistance, capacity-building, and system strengthening (Articles 6, 13) [38]. The agreement further promotes resilient health systems and workforce preparedness via Joint External Evaluations and National Action Plans for Health Security (Articles 6, 12) [37,38]. While education reform and workforce development are indirectly referenced through commitments to research, innovation, and health infrastructure (Article 5), the treaty does not explicitly define curricular modernization or competency standards.

In summary, despite these advances, significant gaps remain. Financing provisions are framed as aspirational rather than binding (Articles 19–22), leaving sustained support uncertain. Mechanisms for regional cooperation rely heavily on political will rather than enforceable obligations, and intellectual property and benefit-sharing provisions remain under negotiation in the PABS annex. Without translating these commitments into binding, well-resourced, and operationally detailed mechanisms, the treaty risks reproducing the very inequities and implementation failures observed during COVID-19.

### 3.4. Results of the Document Analysis

The findings from the scoping review were subsequently mapped against the provisions of the WHO Pandemic Agreement (*WHA78.*) to assess the extent to which the treaty addresses the governance and equity challenges identified in the literature. The correspondence between literature-identified challenges and treaty provisions is summarized in Table 8 below.

A detailed mapping of these linkages, including article-level analysis and degree of coverage for each theme, is provided in Appendix C.

## 4. Discussion

### 4.1. Situating Pandemic Governance Within a Multi-Layered Framework

This discussion interprets the findings through a novel conceptual framework that organizes global pandemic preparedness into four interdependent governance layers: foundational, equity, operational, and feedback layers. These layers function as an interconnected matrix, wherein deficiencies in one dimension can cascade into vulnerabilities in others. For example, shortcomings in the foundational legal architecture, such as the non-binding nature of the International Health Regulations (IHR), can lead to institutional ambiguity, undermine operational effectiveness, and ultimately contribute to inequitable health outcomes.

**Figure 2:**
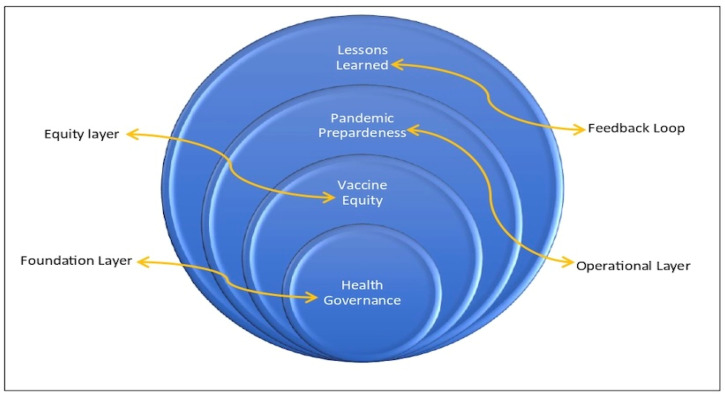
Conceptual Framework of the Discussion.

The foundational layer refers to the legal instruments, treaties, and institutional structures that provide the structural basis for pandemic governance. This includes frameworks such as the International Health Regulations (IHR) and the WHO Pandemic Agreement, which aim to coordinate international responses, define state obligations, and establish mechanisms for reporting and oversight. The equity layer addresses normative and distributive justice, encompassing mechanisms such as intellectual property reform, Access Benefit Sharing frameworks, and financing instruments for low- and middle-income countries. The operational layer reflects how policy is translated into action, including resilient health systems, multisectoral coordination, and preparedness simulations. Finally, the feedback learning layer emphasizes adaptive governance through continuous incorporation of lessons from past outbreaks to refine the other layers. This layered architecture moves beyond descriptive thematization to offer a systemic analytical lens. Figure 2 shows the schematic representation of the multi-layered global health governance framework, illustrating foundational health governance, operational elements of vaccine equity and pandemic preparedness, and a feedback loop for lessons learned to inform continuous improvement across all layers.

### 4.2. Synthesis

The findings from this review line up with the four layers of the conceptual framework, providing both empirical grounding and practical implications.

#### 4.2.1. Foundational Governance Layer

The 57 studies reviewed, published between 2020 and 2024, reveal widespread agreement that existing legal and institutional frameworks are inadequate. Although the International Health Regulations (IHR) form the legal foundation of global health governance, they were consistently critiqued for lacking enforceability and real-time monitoring [27,28]. Scholars therefore advocate for strengthening this legal foundation through treaty-based obligations, independent oversight bodies, and robust accountability mechanisms [25,29], recognizing it as essential to enabling and sustaining pandemic preparedness.

#### 4.2.2. Equity Layer

Equity constitutes the second layer of the governance framework and was a dominant concern across the reviewed literature, particularly in relation to vaccine distribution and intellectual property (IP) regimes. The failure of COVAX to deliver equitable access, achieving only a fraction of its intended vaccine allocations for low- and middle-income countries (LMICs), exposed the structural flaws of voluntary, market-dependent models [39,41]. Specifically, COVAX lacked binding procurement commitments from high-income countries and operated without enforceable obligations on manufacturers, leaving LMICs vulnerable to bilateral deals and supply delays. Intellectual property protections under the TRIPS Agreement further constrained access by limiting technology transfer and local production capabilities. In response, scholars proposed legal mechanisms such as automatic IP waivers during global health emergencies, compulsory licensing for vaccine production, technology transfer, and binding benefit-sharing obligations linked to pathogen access [40,47]. Despite their normative appeal, these proposals face strong geopolitical resistance, particularly from pharmaceutical-exporting nations. Nonetheless, these equity mechanisms are central to correcting systemic imbalances and must be structurally embedded, rather than being aspirational within future governance instruments.

#### 4.2.3. Operational Capacity Layer

At the operational level, the literature reveals a fundamental tension between aspirational health governance principles and their translation into functional preparedness systems. Despite widespread endorsement of integrated strategies, particularly the convergence of Universal Health Coverage (UHC) and Global Health Security (GHS), few health governance systems adequately resolve the disconnect between long-term health system strengthening and short-term emergency response [17,43]. This gap is not merely technical but structural, reflecting deeper misalignments in policy priorities and funding mechanisms. For example, proposals to institutionalize simulation exercises and real-time surveillance are promising, yet often lack sustainable financing and political support, especially in low-resource settings. Similarly, calls to reform health education to include disease modeling, epidemiological surveillance, triage protocols, digital health tools, and predictive analytics face inertia within national curricula, despite their critical role in supporting preparedness and integrated governance [46,49]. Rather than viewing these interventions as discrete technical fixes, the operational layer must be understood as a site where equity goals and legal norms are stress-tested against logistical, institutional, and workforce constraints.

#### 4.2.4. Feedback Loop Layer

The feedback layer reflects the capacity of governance systems to learn from failure and adapt in real time. Rather than relying on reactive adjustments, effective feedback mechanisms should be embedded within institutional and legal frameworks to support anticipatory action and timely course correction. Yet the literature reveals a persistent failure to institutionalize such reflexivity. The COVID-19 experience exposed not only gaps in preparedness but also the absence of structured mechanisms for transforming lessons into systemic reform. The underutilization of the subsidiarity principle illustrates this gap, although frequently cited in global governance discourse, it remains operationally vague and is often sidelined in treaty design [19,44]. Its absence reflects a broader reluctance to share rule-setting and decision-making power with local actors, which undermines both the legitimacy and responsiveness of global instruments. To address this, the feedback layer must move beyond retrospective evaluations toward dynamic learning loops that are institutionalized, inclusive, and legally anchored. Such mechanisms are vital not only for treaty adaptability but also for preserving institutional trust during future crises.

In summary, the multi-layered framework advanced in this review highlights how legal, equity, operational, and adaptive mechanisms must work together for effective pandemic governance. With the WHO Pandemic Agreement formally adopted in May 2025, the analysis offers timely insights into how these governance layers can guide the implementation of the treaty. Bridging high-level principles and practical reforms, the following recommendations identify concrete pathways to strengthen treaty effectiveness and promote equitable, accountable global health systems.

### 4.3. Recommendations and Implementation Roadmap

The recommendations are structured according to the layers of the conceptual framework developed in this review. Each layer, foundational, equity, operational, and adaptive, corresponds to a specific set of actions necessary for strengthening pandemic governance.

#### 4.3.1. Foundational Governance

Revising the International Health Regulations (IHR) to incorporate legally binding compliance mechanisms is essential. This includes mandatory timelines for outbreak reporting and standardized penalties for non-compliance. Furthermore, an independent compliance and monitoring body should be established under the Conference of the Parties (COP), with authority to conduct peer-reviewed audits, publish findings, and recommend corrective actions.

#### 4.3.2. Equity Mechanisms

Operationalizing Access and Benefit-Sharing (ABS) systems should be directly linked to real-time epidemiological data to enable timely and equitable distribution of medical countermeasures. Intellectual property barriers could be mitigated by introducing automatic waiver and compulsory licensing provisions during declared public health emergencies, activated through predefined legal triggers. In addition, regionally distributed research and development (R&D) and manufacturing hubs in LMICs should be established, supported by formal technology transfer agreements and multi-year funding commitments, to reduce structural dependency on high-income countries.

#### 4.3.3. Operational Capacity

Establish a permanent Pandemic Preparedness and Response Fund (PPRF) with needs-based disbursement criteria, prioritizing countries according to vulnerability assessments and internationally recognized preparedness metrics, such as Joint External Evaluations (JEEs) and the Global Health Security Index (GHSI). Tie funding disbursements to annual public expenditure audits and require community-level transparency mechanisms, such as digital dashboards that track fund allocation and use in real time. Invest in resilient primary health care systems that integrate Universal Health Coverage (UHC) and Global Health Security (GHS) objectives, with clear performance indicators. Expand health education to include training in data analytics, disease modeling, emergency triage, and logistics, embedded within medical and public health curricula.

#### 4.3.4. Feedback and Adaptive Governance

Institutionalize the role of regional organizations, such as the Africa CDC, PAHO, and ASEAN, in treaty oversight and localized implementation, applying the principle of subsidiarity to ensure responsiveness to regional contexts. Introduce annual preparedness scorecards based on standardized indicators, including vaccination capacity, surveillance coverage, and supply chain resilience, to benchmark country performance. Require annual national implementation reports, subject to independent verification every three years by an expert review panel, with findings systematically feeding into the treaty’s Conference of the Parties (COP) to inform subsequent revision cycles.

### 4.4. Strengths and Limitations

This study has several limitations inherent to mixed qualitative designs. The scoping review component relied primarily on conceptual and normative literature, reflecting the limited empirical evidence available on global pandemic governance. Consequently, theoretical perspectives may be overrepresented compared to practice-based insights. Additionally, evidence from low- and middle-income countries (LMICs) was underrepresented, highlighting structural publication biases and resource inequities that limit global representativeness.

The document analysis was conducted on the WHO Pandemic Agreement text adopted in May 2025. Key annexes, including the Pathogen Access and Benefit-Sharing (PABS) system, remain under negotiation; therefore, several provisions were coded as Pending. Furthermore, this study does not assess the implementation or effectiveness of the Agreement, which can only be evaluated once it enters into force. Future empirical research, especially incorporating LMIC and regional perspectives, will be essential to evaluate the treaty’s operationalization, financing, and accountability in practice.

Despite these limitations, this study offers one of the earliest integrated analyses linking evidence from global pandemic preparedness literature with the provisions of the newly adopted WHO Pandemic Agreement. By combining a scoping review with qualitative document analysis, this study facilitates systematic triangulation between research-identified governance challenges and treaty commitments. This dual-method design provides a transparent, evidence-to-policy mapping framework and establishes a foundation for future monitoring of implementation and accountability in global health governance.

## 5. Conclusions

This study demonstrates that the WHO Pandemic Agreement, adopted in May 2025, faces critical legal, governance, and equity challenges that must be addressed for it to effectively strengthen global health security. While the treaty represents a significant advancement in international health cooperation, its success depends on the implementation of key provisions, particularly the unresolved Pathogen Access and Benefit-Sharing (PABS) mechanism, and on sustained investment in equitable and resilient health systems.

By combining a scoping review of the global pandemic preparedness literature with a document analysis of the WHO Pandemic Agreement, this two-phase study bridges the gap between evidence and policy. The Multi-Layered Framework developed here highlights the interdependence between legal foundations, equity mechanisms, operational capacity, and adaptive learning as core dimensions of pandemic governance.

These findings illustrate that health outcomes are shaped by the interaction of multiple global systems extending beyond the health sector, encompassing trade, diplomacy, international law, economic policy, and environmental sustainability. This raises a pivotal question: should pandemic preparedness remain confined to a health-sector governance model, or evolve toward a global governance for health approach? The latter offers a more holistic and sustainable pathway, integrating health with broader global governance systems, to achieve equity, accountability, and resilience in future pandemic responses.

## Figures and Tables

**Table 1:** Types of Studies.

Categories	Document/Study Type	Count	(%)
Journals Articles	Commentary article	10	19.2%
	Working Paper	3	5.8%
	Perspective	7	13.5%
	Editorial	4	7.7%
	Viewpoint	13	25.0%
	Opinion	1	1.9%
	Letter To the Editor	1	1.9%
	Narrative review	6	11.5%
	Correspondence	2	3.8%
	Scoping reviews	1	1.9%
Research Papers	Case study	1	1.9%
Background Reports	Comparative health Policy Analysis	1	1.9%
WHO Documents	Research Briefing	2	3.8%

**Table 2:** Geographical distribution of the studies.

Country	Count	Country	Count
United States	9	Germany	1
United Kingdom	10	Hungary	1
Switzerland	8	Argentina	1
Ireland/Austria	3	Saudi Arabia/Japan/Scotland	1
Canada	2	India	1
Australia	2	Malaysia	1
Norway	1	Pakistan	1
Italy	1	China	2
Brazil	1	Ghana	2
Belgium	1	South Africa	1
Netherland	1	Indonesia	1

**Table 3:** Date of Publication.

Year	Count	Observations
2020	8	This is the initial wave of publications, relatively low number due to early stages of the pandemic.
2021	12	Nearly doubled compared to 2020; increased efforts to understand the pandemic; and more data became available.
2022	7	Decrease in publications, period of consolidation, with validation of earlier research findings and focus on specific aspects of the pandemic.
2023	14	A new spike in publications, reaching the highest level in this timeline; as the impact of the pandemic continued, and distribution of vaccines started.
2024	11	Large number of publications indicating sustained interest and activity in pandemic-related research with focus on lessons learned, long-term impacts, and continued efforts to establish global health preparedness.

**Table 4:** Global Health Governance and the Proposed Pandemic Treaty Studies.

Authors and Year	Focus
Behrendt et al., 2022 [14]	Global Health Security Doctrine (GHS)
Fukuda-Parr et al., 2021 [15]	Rethinking Global Health Security
Hosseini, 2023 [16]	Gaps in Global Health Solidarity during COVID-19
Lal et al., 2022 [17]	Universal Health Coverage (UHC) and Global Health Security (GHS) integration
Evaborhene et al., 2023 [18]	Equitable access to medical counter measures (mcm)
Campos-Rudinsky et al., 2024 [19]	Subsidiarity and solidarity
Bustamante et al., 2021 [20]	Global South Perspectives on the Pandemic Treaty
Chattu et al., 2024 [21]	Role of global health diplomacy in the negotiations
Kickbusch & Kökény, 2013 [12]	Global health diplomacy
Cicero & Phelan, 2024 [22]	Equity is a cornerstone for inclusive, pandemic science, where equity and science are balanced.
Emanuel et al., 2022 [23]	Propose an “An ethical framework for global vaccine allocation”
Kickbusch & Holzscheiter, 2021 [24]	Impact of geopolitical dynamics on pandemic treaty
Huang et al., 2024 [25]	China’s Stance on Pandemic Treaty
Gostin et al., 2021 [26]	A safer and equitable world during pandemics.
Gostin & Katz, 2016 [13]	Review IHR Performance
Labonté et al., 2021 [27]	“Pandemic treaty, IHR or Both?”
Solomon, 2022 [28]	Short-Term Negotiation Strategies for Pandemic Treaty
Khor & Heymann, 2021 [29]	Global health governance framework
Vinuales et al., 2021 [30]	“Deep Prevention,” upstream and downstream.
Collins et al., 2020 [31]	Public health priorities for pandemic preparedness
Sheerah et al., 2024 [32]	The Challenges in Developing A Pandemic Treaty
Faviero et al., 2022 [33]	Accountability in Pandemic Preparedness
Schwalbe et al., 2024 [34]	Governance Provisions in the new Pandemic Agreement
Gostin et al., 2023 [35]	Legal and ethical reforms to promote health and justice.
Hampton et al., 2023 [36]	Calls for more effective mechanisms for equitable pandemic response
Berman, et al., 2025 [37]	Challenges In Making the Pandemic Treaty Enforceable

**Table 5:** Vaccine Equity and Intellectual Property Rights.

Author and Year	Focus
Bollyky TJ, et al., 2020 [39]	A framework for the equitable distribution of COVID-19 vaccines.
De Abreu ADJL, et al., 2023 [40]	Challenges in vaccine distribution and equity.
Gurgula O, 2021 [41]	Access to Vaccine Technology and Intellectual Property.
Horng, D.C. 2024 [42]	Vaccine Diplomacy and Equitable Access.
Lie R.K., et al., 2021 [43]	Balancing Global and National Responsibilities.
Geiger S, et al., 2024 [44]	Equitable Global Access to Health Technologies.

**Table 6:** Preparedness Infrastructure and Monitoring Mechanisms.

Author and Date	Focus
Hanbali, 2023 [45]	Inclusion of independent monitoring in the WHO Pandemic Treaty
Jiang et al., 2023 [46]	Benefits and challenges of the pandemic treaty for Least Developed Nations.
El Bcheraoui et al., 2020 [47]	Resilience of health systems
Baker et al., 2022 [48]	Challenging the existing intellectual property regime
Baldé, 2023 [49]	Best practices of civil society engagement in the COVID-19
Drake, 2020 [50]	Simulation guide for enhancing pandemic preparedness.
Patel, 2023 [51]	Gaps in the treaty accord negotiations among the 43 member states
Gandhi et al., 2023 [52]	What the WHO Global Health Accord means for low-income and middle-income countries
Burkle et al., 2020 [53]	The roles of WHO and the International Health Regulations (IHR)
Frieden, et al. 2021 [54]	Would the new treaty on pandemic preparedness help or hinder current and future response efforts.
Schaefer et al., 2023 [55]	Ethical guidelines within the current pandemic accord.
Walckiers et al., 2024 [56]	Developing a treaty with an ethical and scientific foundation.

**Table 7:** Lessons from the COVID-19 Pandemic.

Author and Date	Focus
Sachs, et al. 2022 [57]	The need for an ethical framework focusing on societal needs
Coccolini et al., 2021 [58]	Global experiences and lessons learned from the COVID-19 pandemic
Cunningham and Hopkins, 2023 [59]	To highlight the key lessons from COVID-19
Saqr and WASSON, 2020 [60]	Missed opportunities and the acquired lessons
Titanji et al., 2023 [61]	The positive lessons from the COVID-19 pandemic in low- and middle-income countries
Fawcet, 2021 [62]	Effects of COVID-19 on the Middle East and African countries
Djalante et al., 2020 [63]	The responses of Asian countries to the COVID-19 threat.

**Table 8:** Summary of the Correspondence Between Literature-Identified Governance Challenges and Provisions of the WHO Pandemic Agreement (WHA78.1).

Thematic Domain	Key Challenge Areas (From Literature)	Relevant Articles in WHO Pandemic Agreement	Degree of Coverage	Summary Interpretation
1. Global Health Governance and the WHO Pandemic Agreement	Fragmented coordination; limited transparency; weak legal accountability; insufficient sustainable financing; poor integration of One Health; weak enforcement and regional inclusion	Arts. 3–5, 10–13, 17–18, 31 (+draft PABS Annex, as it still under negotiation).	Partial	Strengthens coordination, financing, and One Health principles, but lacks enforceable accountability, subnational inclusion, and clear compliance mechanisms.
2. Vaccine Equity and Intellectual Property Barriers	Inequitable access to countermeasures; insufficient financing for LMICs; limited technology transfer; weak IP and benefit-sharing mechanisms	Arts. 10, 12–13, 18, 31 (+draft PABS Annex)	Partial/Pending	Advances equity goals via global supply and financing systems but relies on voluntary IP waivers and technology transfer; key PABS modalities remain unresolved.
3. Preparedness Infrastructure and Monitoring Mechanisms	Gaps in compliance and monitoring; limited support for LDNs; weak simulation exercises; under-institutionalized civil-society participation; inadequate financing	Arts. 6, 8, 12–13, 15, 18–22 (+draft PABS Annex)	Partial	Introduces a compliance mechanism and LDN assistance. However, monitoring lacks independence and financing commitments remain aspirational.
4. Lessons from the COVID-19 Pandemic	Delayed reporting; misinformation; weak regional cooperation; inequities in LMIC support; insufficient workforce training and education	Arts. 5–6, 10, 12–13, 18–22 (+draft PABS Annex)	Partial/Full in selected areas)	Improves early-warning and data-sharing duties and embeds One Health; however, education, regional collaboration, and financing provisions lack binding force.

## Data Availability

No new data were created or analyzed in this study. Data sharing is not applicable to this article.

## Appendix A. PRISMA-ScR Checklist

**Section**	**PRISMA-ScR Item**	**Compliance in Manuscript**
Title	Identify the report as a scoping review.	Yes, title states ‘A Scoping Review’
Abstract	Structured summary including background, objectives, eligibility criteria, sources, charting methods, results, and conclusions.	Yes, Structured abstract provided with all elements.
Rationale	Describe rationale for the review in context of what is already known.	Yes, Introduction discusses COVID-19 governance gaps and treaty rationale.
Objectives	Provide explicit review questions and objectives.	Yes, Review question and objectives clearly stated.
Eligibility criteria	Specify characteristics of sources of evidence (e.g., publication years, languages, types).	Yes, Inclusion/exclusion criteria defined.
Information sources	Describe all information sources in the search (databases, registers, websites, etc.) and date last searched.	Yes, PubMed, Scopus, Web of Science, WHO, CDC, UN; searched May–Oct 2024.
Search	Present full electronic search strategy for at least one database.	Yes, Boolean search strings presented in Table 1.
Selection of sources of evidence	State the process for selecting sources (screening, eligibility, included in review).	Yes, Two-stage screening with Covidence described.
Data charting process	Describe methods of charting data from included sources (e.g., forms, multiple reviewers).	Yes, Standardized form in Covidence, 3 reviewers used.
Data items	List and define all variables for which data were sought.	Yes, Publication type, country, date, thematic focus, findings.
Critical appraisal of individual sources	If done, describe methods for assessing risk of bias/quality.	Not conducted, consistent with scoping review methodology.
Synthesis of results	Describe methods of handling and summarizing data.	Yes, Inductive thematic analysis, coding, synthesis into conceptual framework.
Results: Selection of sources	Report numbers screened, assessed, included, with reasons for exclusion.	Yes, Reported with flow diagram (Figure 1).
Results: Characteristics of sources	Present characteristics of each source of evidence.	Yes, Tables by study type, geography, year.
Results: Critical appraisal within sources	If done, present data on risk of bias/quality.	Not applicable in scoping review.
Results: Synthesis of results	Summarize and present charted results.	Yes, Organized into four themes with synthesis tables.
Discussion	Summarize main results, link to review objectives/questions.	Yes, Discussion maps findings to framework and treaty implications.
Limitations	Discuss limitations of the scoping review process.	Yes, Identified limited empirical evidence, HIC bias, timing issues.
Conclusions	Provide interpretation of results, implications for policy/research.	Yes, Conclusion emphasizes treaty gaps and broader governance for health.
Funding	Describe sources of funding and role of funders.	Yes, ‘No external funding’ statement included.

## Appendix B. Search Strategy Applied for Each Database

**Database**	**Search Strategy/Query**	**Limits/Filters Applied**
Web of Science (WOS)	TS = (“pandemic preparedness” OR “global health governance” OR “International Health Regulations” OR “pandemic treaty” OR “pandemic agreement”) AND PY = (2019–2024)	Publication years: 2019/2024
PubMed	(“pandemic preparedness” [Title/Abstract] OR “global health governance” [Title/Abstract] OR “International Health Regulations” [Title/Abstract] OR “pandemic treaty” [Title/Abstract] OR “pandemic agreement” [Title/Abstract]) AND 01/01/2019” [Date—Publication]: “01/05/2019” [Date—Publication])	Language: English; Publication date: 2019/2024
Scopus	TITLE-ABS-KEY (“pandemic preparedness” OR “global health governance” OR “International Health Regulations” OR “pandemic treaty” OR “pandemic agreement”) AND PUBYEAR > 2018 AND PUBYEAR < 2025	Document type: Article; Years: 2019/2024
WHO Global Index Medicus	“Pandemic preparedness” OR “global health governance” OR “pandemic treaty” OR “International Health Regulations”	Years: 2019/2024

## Appendix C. Document Analysis Tables

This annex presents the results of the document analysis of the WHO Pandemic Agreement (WHA78.1), adopted on 20 May 2025. It cross-references governance challenges identified in the scoping review with the corresponding provisions of the Agreement. Each entry summarizes how specific treaty articles address (Fully), partially address (Partially), omit (None), or defer (Pending) identified challenge areas. This mapping provides an integrated evidence-to-policy assessment framework, supporting evaluation of treaty implementation and accountability.

Note: Degree of coverage: Full: (fully addressed); Partial: (addressed with limitations); None: (not addressed); Pending: (awaiting annex finalization).

### Appendix C.1. Theme One: Global Health Governance and the WHO Pandemic Treaty

**Challenge (From Literature)**	**Relevant Text/Excerpt in WHO Pandemic Agreement**	**Article Number(s)**	**Degree of Coverage**	**Notes/Gap Observations**
Weak global coordination and fragmented governance structures	“Each Party shall cooperate and coordinate with international, regional and subregional organizations to strengthen pandemic prevention, preparedness, and response.”	Art. 3 (Purpose and Principles), Art. 4 (Scope and Relationship with Other Instruments)	Partial	Coordination language remains general; no clear mechanism for operational integration across governance levels.
Limited transparency and information-sharing	“Parties shall promptly share information on public health events and ensure timely reporting to WHO.”	Art. 11 (Information Sharing and Notification)	Full	Introduces binding reporting obligations and defines timeliness but lacks verification and enforcement mechanisms.
Insufficient sustainable financing mechanisms	“A Coordinating Financial Mechanism shall be established to mobilize predictable and sustainable resources.”	Art. 13 (Sustainable Financing), Art. 18 (Cooperation and Support)	Partial	Financing principles established, but implementation and monitoring arrangements remain vague.
Lack of enforceable legal accountability	“A Conference of the Parties (COP) shall oversee implementation, review progress, and promote compliance.”	Art. 17 (Implementation and Monitoring) and Art. 31 (COP)	Partial	Oversight depends on voluntary reporting; no independent compliance body or sanctions.
Weak integration of the One Health approach	“Parties shall adopt a One Health approach that integrates human, animal, and environmental health.”	Art. 5 (Objectives), Art. 12 (One Health and Prevention)	Partial	Framework acknowledges One Health but lacks operational standards or intersectoral funding mechanisms.
Limited empowerment of subnational and regional actors	—	—	None	The treaty does not explicitly include regional or local authorities, limiting subsidiarity and contextual responsiveness.
Unclear provisions for equitable data governance and benefit sharing	“A Pathogen Access and Benefit-Sharing (PABS) System shall be established to ensure fair and equitable sharing of benefits.”	Art. 10 (PABS System) + PABS Annex (draft)	Pending	Annex not finalized; data governance and equity metrics remain undefined.
Weak enforcement and compliance measures	“The COP shall adopt procedures and mechanisms to promote compliance.”	Art. 31 (COP)	Partial	No independent monitoring or sanction mechanisms; compliance largely self-reported.

### Appendix C.2. Theme Two: Vaccine Equity and Intellectual Property Barriers

**Challenge (From Literature)**	**Relevant Text/Excerpt in WHO Pandemic Agreement**	**Article Number(s)**	**Degree of Coverage**	**Notes/Gap Observations**
Inequitable access to vaccines and countermeasures	“Each Party shall cooperate to ensure equitable and timely access to pandemic-related products… through a global supply and allocation mechanism.”	Art. 12 (Global Supply System and Allocation Mechanism)	Partial	Establishes principle of equity but lacks measurable allocation criteria or enforcement provisions.
Lack of sustainable financing for manufacturing and distribution in LMICs	“Parties shall mobilize predictable and sustainable financing through bilateral, regional and multilateral mechanisms, including a Coordinating Financial Mechanism.”	Art. 13 (Financing) and Art. 18 (Cooperation and Support)	Partial	Financing is non-binding; resourcing, oversight, and timelines are undefined.
Limited technology transfer and manufacturing capacity	“Parties shall encourage voluntary transfer of technology and know-how to support regional production of pandemic products.”	Art. 18 (Scientific and Technical Cooperation)	Partial	Relies on voluntary mechanisms and existing TRIPS flexibilities; no compulsory licensing triggers or enforcement mechanisms.
Inequitable benefit-sharing for pathogen samples and genetic sequence data	“A Pathogen Access and Benefit-Sharing (PABS) System shall link access to pathogen materials and sequence data with fair and equitable benefit-sharing.”	Art. 10 (Pathogen Access and Benefit Sharing) + PABS Annex (draft)	Pending	Annex under negotiation; benefit types, allocation rules, and triggers not yet specified.
Weak compliance and accountability mechanisms for equity provisions	“A Conference of the Parties (COP) shall monitor implementation and promote compliance with this Agreement.”	Art. 31 (Conference of the Parties)	Partial	COP oversight depends on voluntary reporting; lacks sanctions or independent verification.

### Appendix C.3. Theme Three: Preparedness Infrastructure and Monitoring Mechanisms

**Challenge (From Literature)**	**Relevant Text/Excerpt in WHO Pandemic Agreement**	**Article Number(s)**	**Degree of Coverage**	**Notes/Gap Observations**
Lack of independent monitoring and compliance mechanisms	“A compliance mechanism is hereby established under the authority of the Conference of the Parties (COP) to oversee implementation and promote accountability.”	Art. 19–22 (Compliance and Oversight)	Partial	Introduces compliance framework with self-reporting and peer assessments but lacks independent enforcement powers or sanction mechanisms.
Structural vulnerabilities of Least Developed Nations (LDNs)	“Parties shall provide targeted financial and technical assistance to Least Developed Nations to strengthen health systems and expand equitable access to countermeasures.”	Art. 8 (Equity and Support), Art. 15 (Financial and Technical Assistance)	Partial	Explicit reference to LDNs, but mechanisms for funding delivery, monitoring, and representation in COP remain undefined.
Weak core health system capacities and preparedness planning	“Parties shall develop and maintain core public health capacities, including essential services, preparedness planning, and surveillance.”	Art. 6 (Core Capacities)	Full	Clear obligations aligned with IHR core capacities and JEEs, but resource dependence and uneven national implementation persist.
Intellectual property barriers to equitable access	“A Pathogen Access and Benefit-Sharing (PABS) System shall link pathogen data sharing with equitable access to vaccines, diagnostics, and therapeutics.”	Art. 10 (PABS System) + PABS Annex (draft)	Pending	PABS system under negotiation; lacks defined benefit types, allocation rules, and enforceable technology transfer provisions.
Limited operationalization of civil society participation	“Parties shall promote meaningful participation of civil society organizations and communities in pandemic preparedness and response.”	Art. 12 (Preparedness and Multisectoral Engagement)	Partial	Acknowledges CSO role but remains non-binding; lacks procedural guidelines or inclusion guarantees.
Weak institutionalization of simulation and preparedness exercises	“Parties shall conduct regular simulation exercises to test national and international readiness.”	Art. 12 (Preparedness and Simulation Exercises)	Partial	Provisions are aspirational without standardized implementation procedures or reporting obligations.
Insufficient financing and operational guarantees for equity	“Parties shall promote sustainable and predictable financing for preparedness and equitable access to countermeasures.”	Art. 13 (Sustainable Financing), Art. 18 (Cooperation and Support)	Partial	Financing clauses are general; absence of binding targets or time-bound commitments reduces enforceability.
Unclear mechanisms for regional manufacturing and capacity-building	“Parties shall strengthen regional manufacturing capacity for medical countermeasures.”	Art. 18 (Scientific and Technical Cooperation)	Partial	Recognizes need for regional capacity-building but relies on voluntary cooperation; no clear funding or technology-sharing mandates.

### Appendix C.4. Theme Four: Lessons from the COVID-19 Pandemic

**Challenge (From Literature)**	**Relevant Text/Excerpt in WHO Pandemic Agreement**	**Article Number(s)**	**Degree of Coverage**	**Notes/Gap Observations**
Critical gaps in pandemic response (delayed reporting, weak containment, misinformation)	“Parties shall ensure timely outbreak notification and transparent data sharing.”	Art. 10 (Surveillance and Data Sharing)	Full	Strengthens obligations for transparency and rapid reporting; however, operational enforcement mechanisms and penalties for non-compliance are not specified.
Weak integration of One Health approaches	“Parties shall adopt a multisectoral One Health framework integrating human, animal, and environmental health systems.”	Art. 5, Art. 12 (One Health and Multisectoral Coordination)	Partial	Conceptually embedded but lacks implementation guidance and measurable benchmarks.
Inadequate workforce education and training in public health	“Parties shall promote research, innovation, and capacity building for health systems strengthening.”	Art. 5 (Research and Innovation), Art. 6 (Health Systems Strengthening)	Partial	Indirectly addresses training through R&D and capacity-building but lacks explicit commitments to curricular reform or competency standards.
Structural inequities affecting LMICs	“Parties shall provide financial and technical assistance to strengthen LMIC capacities for preparedness and equitable access to countermeasures.”	Art. 6, Art. 13 (Assistance and Financing)	Partial	Recognizes LMIC support needs, but funding mechanisms are voluntary and not binding.
Weak regional coordination and cooperation mechanisms	“Parties shall cooperate at regional and subregional levels to strengthen preparedness and response capacities.”	Art. 12 (Regional Cooperation), Art. 18 (Technical Collaboration)	Partial	Encourages regional collaboration but lacks institutional structures or accountability frameworks for regional bodies.
Limited attention to misinformation and public trust	“Parties shall ensure accurate, evidence-based risk communication and counter misinformation.”	Art. 10 (Risk Communication and Transparency)	Full	Introduces explicit requirements for transparent communication, though no global monitoring system is defined.
